# Supplementary material for: The Diversified O-Superfamily in Californiconus californicus Presents a Conotoxin with Antimycobacterial Activity
Source: Toxins (Basel). 2019 Feb 20;11(2):128. doi: 10.3390/toxins11020128 (PMC6410186; doi:10.3390/toxins11020128)
Supplement: Supplementary file 1 [file toxins-11-00128-s001.pdf]

Supplementary Materials: The Diversified O-Superfamily in *Californiconus californicus* Presents a Conotoxin with Antimycobacterial Activity

Johanna Bernáldez-Sarabia, Andrea Figueroa-Montiel, Salvador Dueñas, Karla Cervantes-Luévano, Jesús A. Beltrán, Ernesto Ortiz, Samanta Jiménez, Lourival D. Possani, Jorge F. Paniagua-Solís, Jorge Gonzalez-Canudas and Alexei Licea-Navarro

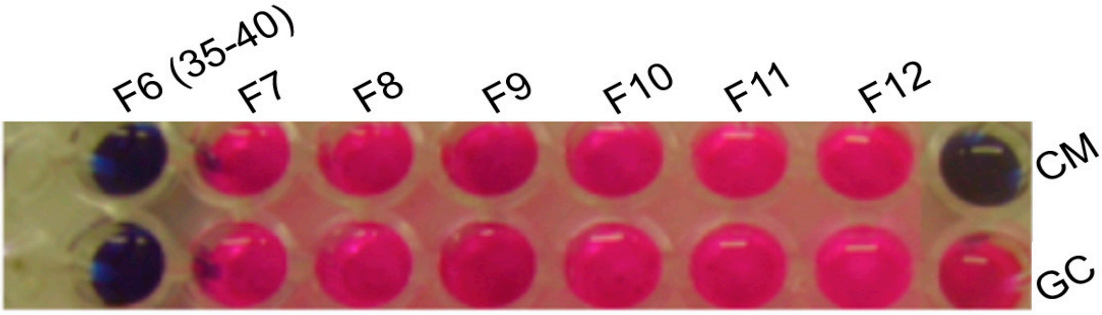

Figure 1. First activity screening of *C. californicus* venom against *M. tuberculosis* H37Rv strain. Twelve RP-HPLC purified fractions were evaluated for their capacity to kill *M. tuberculosis*. Figure shows only the last six fractions, F6 indicates nonmetabolic activity (dead of bacteria, blue color) after adding Blue Alamar reagent. Fractions F7 to F12 shows metabolic activity (live bacteria, pink color). CM it is a well with no bacteria, just media and Blue Alamar reagent; GC it is a well with bacteria and Blue Alamar. Fraction 6 was re-purified and all sub-fractions were tested with the same protocol until a pure peptide was isolated (O1_cal29b). Lower line it is a duplicate of upper line. All fractions were tested at 200 µg/mL.
